# Impact of early PCSK9 inhibitor treatment on heart after percutaneous coronary intervention in patients with STEMI: Design and rationale of the PERFECT II trial

**DOI:** 10.3389/fcvm.2022.1009674

**Published:** 2022-09-23

**Authors:** Jiachun Xia, Xinyue Wang, Jun Zhou, Dong Wang, Yanan Pang, Xin Xu, Zhenchi Sang, Yi Zhang, Junfeng Zhang, Sicheng Wu, Zhengguang Xiao, Lei Hou

**Affiliations:** ^1^Institute of Cardiovascular Diseases, Tongren Hospital, Shanghai Jiao Tong University School of Medicine, Shanghai, China; ^2^Department of Cardiology, Tongren Hospital, Shanghai Jiao Tong University School of Medicine, Shanghai, China; ^3^Collaborative Innovation Centre of Regenerative Medicine and Medical BioResource Development and Application Co-constructed by the Province and Ministry, Guangxi Medical University, Nanning, China; ^4^Department of Cardiology, Shanghai Jiao Tong University Affiliated Chest Hospital, Shanghai, China; ^5^Department of Cardiology, Shanghai Tenth People's Hospital, Tongji University, Shanghai, China; ^6^Department of Cardiology, Shanghai Jiao Tong University School of Medicine Affiliated Ninth People's Hospital, Shanghai, China; ^7^Dental Public Health, The University of Hong Kong Faculty of Dentistry, Hong Kong, China; ^8^Department of Radiology, Tongren Hospital, Shanghai Jiao Tong University School of Medicine, Shanghai, China

**Keywords:** proprotein convertase subtilisin/kexin type 9 (PCSK9), ST-segment elevation myocardial infarction, percutaneous coronary intervention, myocardial salvage index, alirocumab

## Abstract

**Background and aims:**

Primary percutaneous coronary intervention (PPCI) is the most effective treatment strategy for ST-segment elevation myocardial infarction (STEMI). Nevertheless, dysregulated inflammation induced by myocardial reperfusion injury may increase the final infarct size and induce maladaptive myocardial remodeling. Proprotein convertase subtilisin/kexin 9 (PCSK9) inhibitor, as a novel and potent lipid-lowering drug, plays an important role in inflammation. The aim of this study is to investigate whether the early application of PCSK9 inhibitor can increase the myocardial salvage index (MSI) and improve ventricular remodeling in patients with STEMI.

**Design:**

The PERFECT II trial is a prospective, open-label, multicenter, randomized controlled study involving 160 patients with STEMI who are scheduled to undergo PPCI. The eligible patients will be divided into PCSK9 inhibitor group and control group *via* the interactive web response system, at a 1:1 ratio. In the PCSK9 inhibitor group, the PCSK9 inhibitor alirocumab at a dose of 75 mg will be subcutaneously injected immediately after PPCI and administered every 2 weeks thereafter for 3 months based on conventional treatment. In the control group, conventional treatment will be administered. The primary endpoint is MSI, as measured by cardiac magnetic resonance imaging (CMR) at 1 week after PPCI. The secondary endpoints are the peak time of creatine kinase (CK)-MB and troponin I (TnI)/TnT after PPCI; the postoperative fall time of the ST segment on electrocardiography (ECG); the rate of plasma low-density lipoprotein cholesterol (LDL-C) compliance (< 1.4 mmol/L and a reduction of >50% from baseline) at 1, 3, and 6 months after PPCI; infarct size and ejection fraction (EF) measured by CMR at 6 months after PPCI; the occurrence of major adverse cardiovascular event (MACE: a composite of cardiovascular death, non-fatal myocardial infarction, stent thrombosis, repeat revascularization, stroke, and heart failure needed to be hospitalized).

**Conclusions:**

This is the first multicenter study to investigate the effect of early application of the PCSK9 inhibitor alirocumab on MSI in patients with STEMI undergoing PPCI. The findings will provide an opportunity to explore novel ideas and methods for the treatment of acute myocardial infarction.

**Trial registration:**

ClinicalTrials.gov, identifier: NCT05292404.

## Introduction

Primary percutaneous coronary intervention (PPCI) with drug-eluting stent is an effective and common treatment strategy for acute coronary syndrome (ACS), including unstable angina (UA), non-ST-segment elevation myocardial infarction (non-STEMI), and STEMI (1). Over the past decade, the prognosis of acute myocardial infarction (AMI) has improved substantially. However, it is still the leading cause of morbidity and mortality worldwide (2). Myocardial reperfusion is imperative to salvage the ischemic myocardium in patients with STEMI; nevertheless, early restoration of blood flow itself can induce damage to the myocardium by triggering a series of sterile inflammatory responses known as myocardial ischemia-reperfusion injury (3). Crucially, the lack of effective therapy to attenuate local inflammation after myocardial infarction in patients with STEMI makes it necessary to identify a viable target for cardioprotection.

Proprotein convertase subtilisin/kexin 9 (PCSK9), which is mainly secreted by the liver, can increase the concentration of serum low-density lipoprotein cholesterol (LDL-C) by promoting LDL receptor (LDLR) degradation (4). The plasma concentration of PCSK9 is significantly increased in patients with AMI (5). Recent evidence suggests that PCSK9 contributes to inflammation beyond cholesterol regulation. In atherosclerosis progression, PCSK9 activates proinflammatory cytokine production and affects oxidative modifications within atherosclerotic lesions (6, 7). In addition, in animal models of AMI, PCSK9 is up-regulated mostly in the region bordering the infarct area and determines the development of infarct size, cardiac function, and autophagy (8). Additionally, the concentration of serum PCSK9 is associated with the future risk of cardiovascular disease (9). PCSK9 inhibitors like alirocumab are frequently performed to reduce the concentration of LDL-C and lower the rate of adverse cardiovascular events (10). Moreover, application of alirocumab and the anti-PCSK9 AT04A vaccine in APOE^*^3Leiden.CETP transgenic mice reduces vascular inflammation and atherosclerotic lesions (11, 12). Our team found that PCSK9 knockout mice showed ameliorated myocardial inflammation and heart failure after myocardial infarction.

We recently conducted a randomized pilot study (PERFECT trial, NCT04731105) in 20 patients with anterior myocardial infarction accompanied by chest pain that began within 24 h. In this study, a single 75-mg dose of alirocumab was subcutaneously injected immediately after PPCI and lead to a trend toward an increase in the myocardial salvage index (MSI) compared with the control group (0.50 ± 0.16 vs. 0.41 ± 0.15; *p* = 0.21).

To further confirm the results of the PERFECT trial, we designed this multicenter, randomized controlled study to investigate whether early application of alirocumab in patients with STEMI can increase the MSI 1 week after injection and decrease the infarct size 6 months later. The MSI, as assessed by cardiac magnetic resonance imaging (CMR) with late gadolinium enhancement, is considered to be the gold-standard method to assess of infarct size (13). Thus, we will use the MSI, instead of infarct size, as the primary endpoint in this study.

## Methods

### Study objectives

The primary objective of this study is to explore whether the early application of the PCSK9 inhibitor alirocumab after successful PPCI increases the MSI, limits infarct size, and improves ventricular remodeling in patients with STEMI.

### Study design

**Figure 1:**
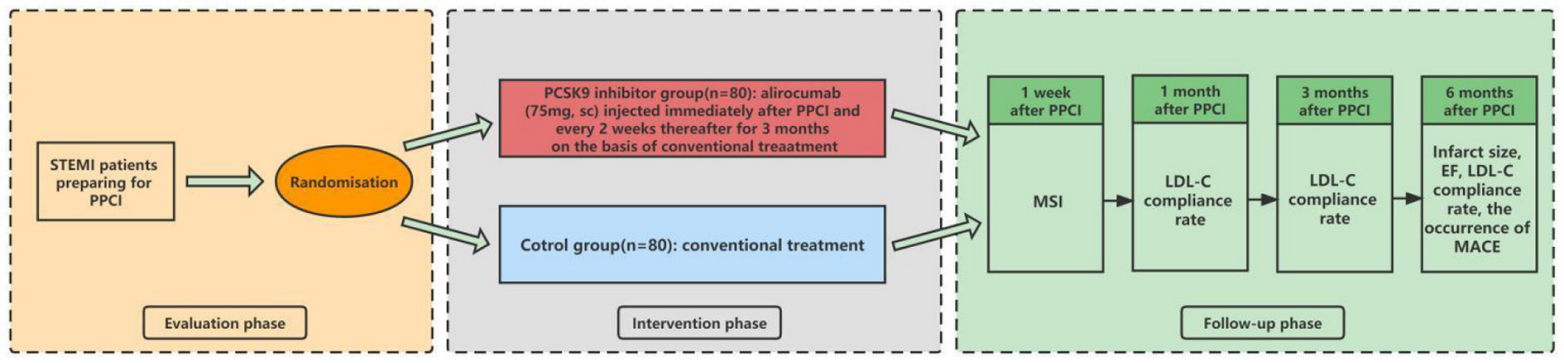
Study flow chart. STEMI, ST-segment elevation myocardial infarction; PPCI, primary percutaneous coronary intervention; sc, subcutaneous; MSI, myocardial salvage index; LDL-C, low-density lipoprotein cholesterol; EF, ejection faction; MACE, major adverse cardiovascular event.

The PERFECT II trial is a multicenter, prospective, open-label, randomized controlled clinical trial. The centers of this study include Tongren Hospital, Chest Hospital, Tenth People's Hospital, and Ninth People's Hospital in Shanghai. STEMI patients with an onset of within 24 h who are scheduled to undergo PPCI will be randomized in a 1:1 fashion to receive alirocumab (PCSK9 inhibitor treatment group) or conventional treatment (control group); each group will include 80 eligible patients. The study flow chart is shown in Figure 1.

### Study population

Patients with STEMI who are scheduled to undergo PPCI will be evaluated for enrollment. The diagnosis of STEMI will be based on 2017 European Society of Cardiology guidelines (14). The onset of STEMI must be within 24 h, and participants must be aged 18 to 80, inclusive but not 18. Importantly, written informed consent will be obtained before enrollment. Patients who meet these enrollment criteria will also be judged against the exclusion criteria. For example, patients who are allergic to PCSK9 inhibitors and aspirin (which is an important component of conventional treatment) will be excluded. To exclude the effect of pregnancy on the results of this trial and the effect of drugs on pregnant women, patients who are pregnant, breast feeding, or planning to become pregnant in the next 6 months will not be considered for inclusion. To maximize the completeness of follow-up, we intend to select patients with a life expectancy of >6 months; therefore, the following patients will be excluded: patients with severe hepatic or renal insufficiency and patients with malignant tumors. Patients who have undergone previous revascularization or treatment with PCSK9 inhibitors previously will also be excluded also. Meanwhile, patients who are deemed unsuitable for participation in this study by the investigator will not be eligible for inclusion. In all cases, the final decision will be made by the investigator based on clinical factors and a review of the initial angiography results. The detailed inclusion and exclusion criteria are presented in Table 1.

**Table 1:** Study inclusion and exclusion criteria.

**Inclusion criteria**
1. Patients aged 18 to 80, inclusive but not 18
2. STEMI patients with an onset of within 24 h who are scheduled to undergo PPCI with drug-eluting stent
3. Patients who have provided written informed consent
**Exclusion criteria**
1. Patients who are allergic to PCSK9 inhibitors and aspirin
2. Patients with multivessel disease who are scheduled to undergo elective intervention within 6 months
3. Patients who have undergone revascularization or treatment with PCSK9 inhibitors previously
4. Women who are known to be pregnant, breastfeeding, or preparing for pregnancy in the following 6 months
5. Life expectancy of < 6 months
6. Severe hepatic or renal dysfunction (ALT >5-fold ULA, eGFR < 15 mL/min/1.73 m^**2**^)
7. Active malignant tumor
8. Patients considered to be unsuitable for participation in this study by the investigator

### Randomization

Randomization procedures will be conducted strictly before PPCI. Eligible patients will be randomized 1:1 to the PCSK9 inhibitor group or the control group and stratified by age (≤ 60/>60; 2 categories), gender (female/male; 2 categories), time from symptom onset (< 3/3–24 h; 2 categories) using the interactive web response system (IWRS) after providing written informed consents.

### Interventions and follow-up

After grouping, enrolled patients will undergo PPCI with new-generation drug-eluting stents. Immediately after PPCI, patients grouped in the PCSK9 inhibitor group will be injected with alirocumab subcutaneously at a dose of 75 mg after the PPCI, followed by injections at the same dose every 2 weeks for 3 months on the basis of conventional treatment. Patients in the control group will receive conventional treatment in accordance with the published guidelines, including dual anti-platelet therapy and statins (15). Patients will be evaluated at 1 week and at 1, 3, and 6 months after the index PPCI, and the occurrence of clinical events will be monitored. As the primary endpoint, the MSI will be measured by CMR at 1 week after PPCI. As the key secondary endpoints, the infarct size and ejection fraction (EF) will also be measured by CMR at 6 months after PPCI. Additionally, the peak time of creatine kinase (CK)-MB and troponin I (TnI)/TnT, as well as the postoperative fall time of the ST segment on electrocardiography (ECG) will be recorded. Plasma LDL-C compliance rate (< 1.4 mmol/L and reduction >50% of baseline) and the occurrence of major adverse cardiovascular event (MACE: a composite of cardiovascular death, non-fatal myocardial infarction, stent thrombosis, repeat revascularization, stroke, and heart failure needed to be hospitalized) will be recorded at 1, 3, and 6 months after PPCI (16). Besides, medications at discharge will be recorded, especially those that affect cardiac remodeling. The investigators will follow up with the patients by office visits or telephone contact. The data collected during all follow-ups will be submitted to the original documents. If any patient is admitted to a non-research hospital again, every effort will be made to obtain the original data from the hospital. For all re-infarctions, ECG and myocardial enzyme data will be obtained and recorded. The following events will lead to termination of the patient's follow-up: (1) death; (2) voluntary withdrawal; (3) evacuation of patients by the investigators based on clinical manifestations; and (4) loss to follow-up (informal withdrawal). The follow-up schedule is shown in Figure 1 and Table 2.

**Table 2:** Timetable of activities planned during the study.

**Time point**	**Before operation**	**Follow-up**			
		**1 week**	**1 month (±10 days)**	**3 months (±20 days)**	**6 months (±20 days)**
Informed consent	×				
Demographics/medical history	×				
Randomization	×				
Coronary angiography	×				
Fasting blood glucose	×				
Blood routine test	×	×			
Creatinine, urea nitrogen	×	×			
Electrocardiography	×	×			
CK, CK-MB, TnI/TnT	×	×			
Echocardiography	×	×			×
TC, LDL-C, TG	×	×	×	×	×
CMR		×			×
Medication		×	×	×	×
Drug side effects		×	×	×	×
MACE		×	×	×	×

CK, creatine kinase; CK-MB, creatine kinase isoenzymes; TnI/TnT, troponin I/troponin T; TC, total cholesterol; LDL-C, low-density lipoprotein cholesterol; TG, triglyceride; CMR, cardiac magnetic resonance imaging; MACE, major adverse cardiovascular event.

### Study endpoints

The primary endpoint is MSI measured by CMR at 1 week after the index PPCI. The secondary endpoints are the peak time of CK-MB and TnI/TnT after PPCI; the postoperative fall time of the ST segment on ECG; the rate of plasma LDL-C compliance (< 1.4 mmol/L and a reduction of >50% from baseline) at 1, 3, and 6 months after PPCI; infarct size and EF measured by CMR at 6 months after PPCI; the occurrence of MACE.

### Sample size calculation

Referring to our prior small sample size study, the MSI was higher in the PCSK9 inhibitor group (mean: 0.50; standard deviation [SD]: 0.16) than in the control group (mean: 0.41; SD: 0.15). To guarantee 90% power of the study at a significance level of 0.05, at least 64 patients per group will be needed. Considering a dropout rate of 20%, the required sample size is 160 (80 patients in each group). PASS 15 was used to calculate the sample size.

### Statistical analysis

Continuous variables (including the primary endpoint) will be expressed as mean (SD) or median (interquartile rage) and will be compared using the *t*-test or the Mann-Whitney *U* test, as appropriate. Categorical variables will be expressed as counts and percentages and compared using the χ2 test or Fisher's exact test, as appropriate. We plan to assess the consistency of the treatment effect on the primary endpoint among three pre-specified subgroups that will be analyzed individually and then in a multivariate model. The following subgroup analyses are planned: age: < 60 years vs. ≥60 years; sex: female vs. male; duration from symptom onset to study drug infusion: < 3 h vs. 3–24 h. Safety analyses will include tabulation of the type and frequency of all adverse events and severe adverse events. All primary and secondary endpoints will be analyzed on an intention-to-treat basis (all patients will be analyzed as part of their designated treatment group) and protocol-based premise (analyses will only be performed as part of their designated treatment group when the patient received their designated treatment). For the intention-to-treat analysis, all patients who have provided written informed consent and who have been randomized in the study will be included in the analysis sample, regardless of whether they have received the correct treatment or whether there is crossover. For the protocol-based analysis, only registered patients who received the specified treatment will be included in the analysis sample. Patients who are lost to follow-up will be eliminated at the time of the last known contact. *P* values and confidence intervals will be two-sided with a significance level of 0.05.

### Ethics and dissemination

This trial has been approved by the ethics committee of Tongren Hospital, Shanghai Jiao Tong University School of Medicine (2022-006-01). The study protocol and informed consent prior to patient participation will be approved by the ethics committee. No changes to the protocol or informed consent will be allowed without the approval of the ethics committee. As required by the ethics committee, the investigator will report the progress of the study until completion. Additionally, any protocol amendments and associated informed consent changes will be submitted to the ethics committee and must be approved in writing prior to implementation. Enrolled patients can withdraw from the trial at anytime during the process of the study. The privacy of patients will be strictly maintained during and after the study. Questions related to privacy and secrecy are listed in the informed consent form. All data will only be accessed by the study staff and will be kept strictly confidential. In addition, the data will be protected in locked cabinets at the participating clinical centers. An independent data monitoring committee (IDMC) will review the security data, including data on death, myocardial infarction, stroke, and other adverse events. The IDMC has the right to recommend suspension of enrollment or termination of the research based on safety considerations. The executive committee will make a final decision on the early termination of the study based on the recommendations.

The results will be disseminated at several research conferences and as published articles in peer-reviewed journals after approval from the study sponsors.

## Discussion

Although early revascularization by PPCI is essential for myocardial myocardium, large infarctions frequently occur in patients with STEMI. Consequently, mortality remains high, and many patients develop complications, such as heart failure, after STEMI. Local and severe sterile inflammation is one of the main causes of ischemia–reperfusion injury (17). Despite some prior studies not meeting the expected goals (18–20), depressing inflammation directly or indirectly in the early stage of STEMI is a feasible strategy to protect the myocardium and limit infarct size to improve outcomes.

PCSK9 mainly regulates the concentration of LDL-C by degrading the LDLR; thus, PCSK9 inhibitors have become a novel effective therapeutic strategy for lipid management in patients with hypercholesterolemia and ACS. Recent studies have indicated that PCSK9 also plays a role in inflammation. PCSK9 also mediates cardiomyocyte autophagy (8) and aggravates inflammation through the Toll-like receptor 4/nuclear factor-κB (21) and lipoprotein receptor-related protein 1 (22) pathways. Conceivably, PCSK9 may be a new target for the treatment of AMI. Recent studies have shown that PCSK9 inhibitors can reduce cardiovascular events and attenuate the risk of type 2 MI in patients with ACS (23, 24). Thus, early application of PCSK9 inhibitors may be an effective treatment strategy for patients with STEMI.

In the PERFECT II trial, we expanded the sample size, inclusion criteria, and the number of clinical centers on the basis of the PERFECT trial. Therefore, the PERFECT II trial will provide better evidence on the impact of early application of the PCSK9 inhibitor alirocumab on MSI and ventricular remodeling in patients with STEMI after PPCI.

## Author contributions

LH and JX co-designed the study. SW designed the statistical plan. JX, XW, JZho, DW, YP, XX, ZS, YZ, and JZha screen and enroll participants and arrange informed consent of the participants. ZX is responsible for CMR testing. LH, JX, and XW wrote the manuscript. All authors critically reviewed and approved the final manuscript.

## Funding

This work was supported by the Xinxin Heart Foundation from China Cardiovascular Association (2021-CCA-ACCESS-73).

## Conflict of interest

The authors declare that the research was conducted in the absence of any commercial or financial relationships that could be construed as a potential conflict of interest.

## Publisher's note

All claims expressed in this article are solely those of the authors and do not necessarily represent those of their affiliated organizations, or those of the publisher, the editors and the reviewers. Any product that may be evaluated in this article, or claim that may be made by its manufacturer, is not guaranteed or endorsed by the publisher.
